# Sleep deprivation and suicide risk among minoritized US adolescents

**DOI:** 10.1186/s12888-023-05074-3

**Published:** 2023-08-31

**Authors:** Victoria A. Joseph, Noah T. Kreski, Katherine M. Keyes

**Affiliations:** Department of Epidemiology, Columbia University Mailman School of Public Health, 722 W 168th St, New York, NY 10032 USA

**Keywords:** Suicidality, Sleep duration, Adolescents, Minoritized youth

## Abstract

**Objectives:**

To assess (1) the prevalence of suicide ideation/behavior among adolescents with short sleep by race/ethnicity and (2) the association between sleep duration and suicidal ideation and behavior among American youth by race/ethnicity from 2007 to 2019.

**Methods:**

Data were drawn from the Youth Risk Behavior Surveillance System (YRBSS). Logistic regression analyses were used to assess the relationship between sleep duration and suicidal ideation/behavior.

**Results:**

Overall, suicide ideation/behavior increased among U.S. adolescents of all racial groups from 2007 to 2019. Adjusting for race/ethnicity, sexual identity, age, sex, substance use, trauma, and bullying, those with short sleep had approximately twice the odds [OR: 1.92 (95% CI: 1.65, 2.23)] of suicide ideation/consideration compared to those with long sleep. Stratified analyses indicated that Black students with short sleep had higher odds of making a suicide plan (OR = 1.51, 95% C.I.: 1.27, 1.79) compared with Black students with long sleep. A similar pattern was observed across other racial/ethnic groups (e.g., Hispanic: (OR = 1.74, 95% C.I.: 1.53, 1.97).

**Conclusion:**

Emphasis on suicide interventions is of the essence, especially with increasing rates. Sleep duration significantly predicts suicide risk among all adolescents. Additional research is needed to assess factors that predict suicide among minoritized adolescents, specifically Black and Hispanic adolescents.

**Supplementary Information:**

The online version contains supplementary material available at 10.1186/s12888-023-05074-3.

## Introduction

Suicide rates among adolescents are increasing, and differentially affecting youth and young adults racialized with minority status [1–3]. Emerging disparities among minoritized groups suggest a need to identify potential risk factors that vary in prevalence and strength by racialized group membership. Sleep health is one such factor that is associated with numerous adverse health issues such as obesity [4, 5], depression [6], substance use, and suicide in adolescents [7] as well as cardiovascular disease [8], cognitive problems, poor mental health, and increased mortality risk in adulthood [6, 7, 9, 10]. Given that most U.S. adolescents do not achieve the recommended amount (7–9 h) of nightly sleep [7, 11], and that prior studies have documented racialized disparities in sleep sufficiency and length, sleep as a risk factor for suicidal behaviors is an important component of adolescent health to understand in greater detail.

Sleep disturbances are a known risk factor for suicidal ideation and mental health [12, 13], yet less is known about the associations among minoritized individuals. In 2019, 77% of U.S. high school students reported sleeping for less than 8 h on most nights [14]. Sleep is hypothesized to impact suicidal ideation due to neurobiological factors such as the impact of sleep on serotonin and other factors involved in mood regulation, as well as the impact of nightmares and other components of short and distributed sleep [15]. The relationship between suicide behavior and sleep duration may be different between racial groups, given that Black individuals are more likely to report short sleep when compared to their White counterparts [16]. Short sleep duration is associated with increased suicide ideation among White individuals but only marginally associated with suicide ideation among Black individuals [17], warranting further investigations.

Few studies assess the impact of sleep duration on suicidal ideation and behavior among minoritized youth, testing for differential associations by race. With the recent rapid increases in suicide rates among minority youth, raising concern about the impact of sleep deprivation on suicide ideation and behavior highlights the necessity for suicide-related interventions among racialized adolescents. The current study seeks to assess (1) the prevalence of suicide ideation/behavior among adolescents with short sleep by race/ethnicity and (2) the association between sleep duration and suicidal ideation and behavior among American youth by race/ethnicity.

## Participants and methods

### Study population

The Youth Risk Behavior Surveillance System (YRBSS) is a series of cross-sectional studies initiated in 1990 and released every two years. The Youth Risk Behavior Survey (YRBS) is reviewed by the Centers for Disease Control and Prevention’s Institutional Review Board and designed to protect students’ privacy; local parental permission procedures were also followed. The YRBSS includes school-based surveys of representative samples of high school students from the 9th to 12th grade. Participation in the survey is anonymous and voluntary. This study uses de-identified and publicly available data from the YRBS Combined Datasets which include nationwide YRBS surveys conducted from 2007 to 2019, given that sleep duration was queried beginning in 2007. For our analyses, we focus on adolescents with relevant exposure, outcome, and covariate data. The total sample contains data on 103,525 adolescents. Supplemental Tables 1–4 present summary statistics of study participants by two categories of suicide ideation/behavior.

### Measures

#### Outcome variables- suicide attempt, ideation, plan, and injurious attempt

There were four outcome variables: suicide attempt, suicide ideation, suicide plan, and injurious suicide attempt. Survey items used included: “During the past 12 months, how many times did you actually attempt suicide” (suicide attempt); “During the past 12 months, did you seriously consider attempting suicide” (suicidal ideation/considered suicide); “During the past 12 months, did you make a plan about how you would attempt suicide?” (suicide plan); “If you attempted suicide during the past 12 months, did any attempt result in an injury, poisoning or overdose that had to be treated by a doctor or nurse?” (injurious suicide attempt). All variables were dichotomized as 1 vs. 0. Although all outcome variables were significantly correlated with each other (with Pearson Correlation Coefficients (PCC) > = 0.54), except for injurious suicide attempt (PCCs ranging from − 0.32 to − 0.33), we were interested in the impact of sleep duration on each suicide outcome as each of those outcomes indicates specific information.

#### Main explanatory variable- sleep duration

The American Academy of Sleep Medicine (AASM) recommends that children aged 6–12 years sleep 9–12 h per night and adolescents aged 13–18 years sleep 8–10 hours [18]. In this study, short sleep for adolescents was defined as less than 8 h per night, consistent with recommendations and other analyses [6]. Sleep duration was dichotomized as less than 8 h of sleep and 8 h or more based on the AASM recommendations using a survey item asking “On an average school night, how many hours of sleep do you get? A. 4 or less hours, B. 5 hours, C. 6 hours, D. 7 hours, E. 8 hours, F. 9 hours, G. 10 or more hours”.

#### Covariates

We included covariates based on their noted impact on suicide outcomes and sleep in the literature [19–21]. We included a bridged 4-level variable for race/ethnicity (Non-Hispanic White, Non-Hispanic Black or African American, Hispanic/Latino and an overarching “All other races” variable comprised of American Indian/Alaska Native, Asian, Native Hawaiian/Other Pacific Islander and Multiple Races- Non-Hispanic)), sexual identity (heterosexual/straight, gay or lesbian, bisexual and not sure), age (for our analyses we combined those who are 12 years old or younger,13, 14, and 15 vs. those 16 years or older), sex, substance use (alcohol use, marijuana use, and cocaine use), bullying (on school property or online), and sexual trauma as covariates. We created an overall binary trauma variable including experiences of sexual assault (occurrences of forced sexual contact) and dating violence (physically hurt by romantic partner). Experiences of sexual assault were created using the item: “Have you ever been physically forced to have sexual intercourse when you did not want to?”. Response options were binary yes or no. Dating violence was created using the items: “During the past 12 months, how many times did someone you were dating or going out with force you to do sexual things that you did not want to do? (Count such things as kissing, touching, or being physically forced to have sexual intercourse.)”, response options were ordinal reporting from 0 to 6 or more times, and “During the past 12 months, how many times did someone you were dating or going out with physically hurt you on purpose? (Count such things as being hit, slammed into something, or injured with an object or weapon.)”. Response options included ‘I did not date or go out with anyone during the past 12 months’ as well as ordinal responses of 0 to 6 or more times.

#### Statistical analysis

We examined the prevalence of suicide ideation/behavior among adolescents with short sleep by race/ethnicity. Descriptive statistics were assessed for sleep duration and covariates by suicide ideation/behavior outcomes. Logistic regression was used to assess the relationship between sleep duration and suicidal ideation/behavior. Models were then assessed for multiplicative interactions between sleep duration and race/ethnicity. All analyses were conducted using SAS 9.4 including strata, cluster, weights, and domain statements per YRBS documentation and RStudio. We used a significance level of 0.05 for all statistical tests.

## Results

Supplemental Tables 1–4 show descriptive results among suicide outcome variables (suicide attempt, injurious suicide attempt, suicide ideation/consideration, suicide plan).

**Fig. 1:**
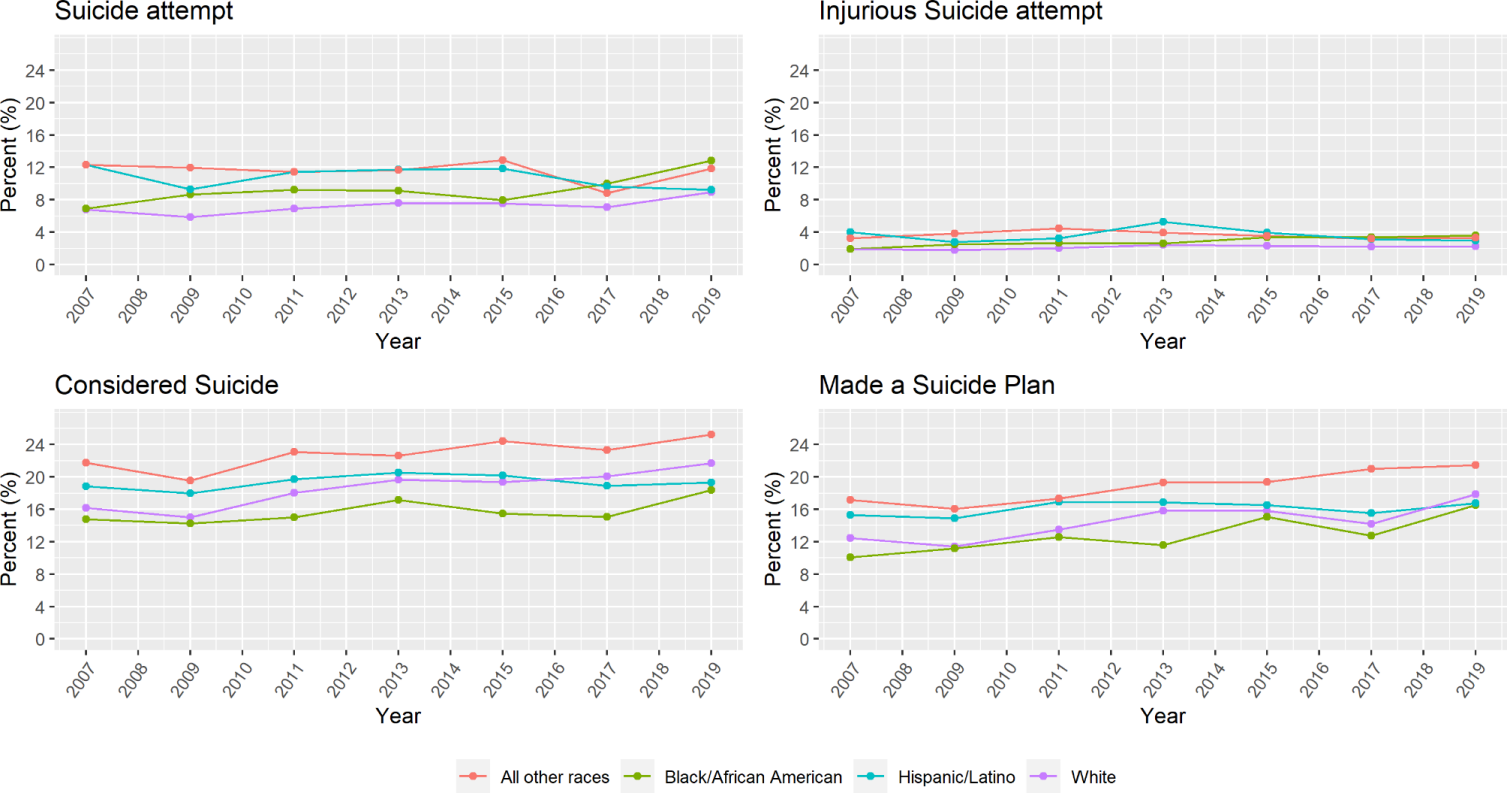
Prevalence of suicide by race/ethnicity among US youth with short sleep, 2007–2019

Figure 1 shows the prevalence of suicide ideation/behavior among US adolescents with short sleep over time by race/ethnicity. Overall, suicide ideation/behavior increased among U.S. adolescents of all racial groups from 2007 to 2019. Suicide attempts sharply increased between 2015 and 2019 among Black adolescents whereas suicide ideation remained highest among adolescents in the “all other races category” throughout the entire study record. Supplemental Fig. 1 shows the overall suicide trends overtime among U.S. adolescents across all racial groups.

Adjusting for race/ethnicity, sexual identity, age, sex, substance use, trauma, and bullying, those with less than 8 h of sleep (short sleep) had approximately twice the odds [OR: 1.92 (95% CI: 1.65, 2.23)] of suicide ideation/consideration compared to those with 8 or more hours of sleep (long sleep). Other outcomes (attempts, injurious attempts, plan) followed a similar pattern as suicide ideation at a similar magnitude (Table 1). Supplemental Table 5 shows unadjusted associations between suicidal ideation/behavior and sleep duration.

**Table 1:** Multivariable logistic regression assessing the relationship between sleep duration and suicide adjusting for race/ethnicity, sexual identity, age, sex, substance use variables, trauma, and bullying

	Suicide attempt vs. no OR (95% CI)	Considered suicide vs. no OR (95% CI)	Made a suicide plan vs. no OR (95% CI)	Injurious suicide attempt vs. no OR (95% CI)
Sleep duration (ref=8 h of sleep or more)				
Less than 8 h of sleep	1.77 (1.34, 2.35)	1.92 (1.65, 2.23)	1.82 (1.53, 2.16)	2.16 (1.34, 3.47)
Race (ref = White)				
All other races	1.99 (1.50, 2.63)	1.38 (1.17, 1.62)	1.42 (1.19, 1.68)	1.63 (0.89, 3.00)
Black or African American	1.41 (1.01, 1.95)	0.79 (0.64, 0.96)	0.88 (0.68, 1.13)	2.36 (1.21, 4.62)
Hispanic/Latino	1.55 (1.21, 1.97)	0.96 (0.84, 1.09)	1.06 (0.93, 1.20)	1.57 (0.97, 2.52)
Sexual identity (ref = heterosexual)				
Bisexual	4.19 (3.26, 5.40)	4.29 (3.61, 5.08)	4.45 (3.67, 5.41)	3.01 (1.72, 5.26)
Gay or Lesbian	2.15 (1.13, 4.11)	3.60 (2.55, 5.08)	3.67 (2.57, 5.25)	2.87 (1.36, 6.04)
Not sure	1.43 (0.97, 2.11)	2.30 (1.79, 2.96)	2.57 (2.06, 3.21)	1.23 (0.60,2.50)
Age (ref=>= 16 years)				
12–15 years	1.43 (1.21, 1.70)	1.07 (0.95, 1.21)	1.05 (0.93, 1.18)	1.41 (0.97, 2.06)
Sex (ref = male)				
Female	1.22 (0.96,1.55)	1.49 (1.31, 1.71)	1.37 (1.22, 1.54)	1.37 (0.86, 2.18)
Alcohol use (ref = no drink in past 30 days)				
Drink at least one day in the past 30 days	1.53 (1.23, 1.92	1.36 (1.19, 1.56)	1.46 (1.24,1.71)	1.92 (1.27, 2.88)
Marijuana use (ref = no marijuana uses over past 30 days)				
Marijuana use at least once in the past 30 days	1.72 (1.36,2.18)	1.58 (1.35, 1.86)	1.51 (1.29, 1.77)	1.49 (0.94, 2.35)
Cocaine use (ref = no uses of any form of cocaine in life)				
Used a form of cocaine at least once in life	1.60 (1.06, 2.44)	1.62 (1.26, 2.09)	1.27 (0.94, 1.73)	3.58 (2.16,5.91)
Trauma (ref = no)				
Yes	5.35 (3.30, 8.67)	4.23 (2.70, 6.61)	3.68 (2.41, 5.62)	5.56 (3.14, 9.83)
Bullying (ref = no)				
Yes	4.80 (3.66, 6.30)	4.13 (3.48, 4.90)	3.68 (3.07, 4.42)	4.73 (2.97, 7.53)

Logistic regression models assessing interactions between sleep and racial/ethnic group show significant results (Supplemental Table 6). Sleep significantly interacted with race/ethnicity; in particular there were significant interactions between sleep duration and Black or African American race, and sleep duration and Hispanic/Latino ethnicity, in predicting suicide attempt (p = 0.001 and 0.01 respectively) and considering suicide (p = 0.0003 and 0.04 respectively). Furthermore, there was a significant interaction between sleep duration and Black or African American race in predicting making a suicide plan (p = 0.02). These interactions indicate that the relationship between sleep and suicidal behavior significantly varies for these groups for a range of suicide outcomes. Table 2 presents odds ratios and 95% confidence intervals from adjusted logistic regression models. The direction of the odds ratios for the interactions generally was that the relationship was more diminished and of smaller magnitude among Hispanic/Latino adolescents when predicating all suicide outcomes and higher among Black or African American and adolescents in the “all other races” category when predicting making a suicide plan. For example, Black or African American adolescents with short sleep had increased odds of making a suicide plan (OR = 1.23, 95% C.I.: 0.69, 2.20) compared with White adolescents.

**Table 2:** Multivariable logistic regression assessing the relationship between sleep duration and suicide adjusting for race/ethnicity, sexual identity, age, sex, substance use variables, trauma, bullying and interactions with sleep duration and race/ethnicity

	Suicide attempt vs. no OR (95% CI)	Considered suicide vs. no OR (95% CI)	Made a suicide plan vs. no OR (95% CI)	Injurious suicide attempt vs. no OR (95% CI)
Sleep duration (ref=8 h of sleep or more)				
Less than 8 h of sleep	2.03 (1.38, 2.98)	1.92 (1.57, 2.34)	1.73 (1.36, 2.20)	3.13 (1.28, 7.65)
Race/ethnicity (ref = White)				
All other races	3.09 (1.55, 6.18)	1.23 (0.80,1.89)	0.89 (0.55, 1.44)	1.96 (0.44, 8.79)
Black or African American	1.38 (0.51, 3.73)	0.87 (0.59, 1.29)	0.74 (0.43, 1.26)	2.96 (0.75, 11.70)
Hispanic/Latino	1.76 (1.05, 2.96)	0.98 (0.72, 1.32)	1.13 (0.81, 1.58)	2.70 (0.88, 8.26)
Sexual identity (ref = heterosexual)				
Bisexual	4.18 (3.26, 5.35)	4.30 (3.63, 5.10)	4.48 (3.68, 5.44)	3.02 (1.72, 5.280
Gay or Lesbian	2.19 (1.13, 4.22)	3.60 (2.55, 5.08)	3.66 (2.57, 5.21)	2.90 (1.36, 6.17)
Not sure	1.43 (0.97, 2.13)	2.32 (1.81, 2.99)	2.59 (2.08, 3.24)	1.22 (0.59, 2.51)
Age (ref=>= 16 years)				
12–15 years	1.43 (1.20, 1.70)	1.07 (0.95, 1.21)	1.05 (0.93, 1.19)	1.40 (0.96, 2.04)
Sex (ref = male)				
Female	1.23 (0.97, 1.56)	1.48 (1.30, 1.68)	1.36 (1.22, 1.53)	1.39 (0.87, 2.21)
Alcohol use (ref = no drink in past 30 days)				
Drink at least one day in the past 30 days	1.53 (1.22, 1.92)	1.36 (1.19, 1.56)	1.46 (1.25, 1.71)	1.90 (1.26, 2.86)
Marijuana use (ref = no marijuana uses over past 30 days)				
Marijuana use at least once in the past 30 days	1.72 (1.36, 2.18)	1.58 (1.34, 1.86)	1.51 (1.28, 1.77)	1.50 (0.95, 2.37)
Cocaine use (ref = no uses of any form of cocaine in life)				
Used a form of cocaine at least once in life	1.55 (1.01, 2.38)	1.68 (1.30, 2.16)	1.30 (0.96, 1.75)	3.55 (2.15, 5.89)
Trauma (ref = no)				
Yes	5.09 (3.12, 8.30)	4.55 (2.99, 6.92)	3.77 (2.51, 5.68)	5.46 (3.08, 9.66)
Bullying (ref = no)				
Yes	4.79 (3.64, 6.29)	4.15 (3.51, 4.91)	3.77 (2.51, 5.68)	4.72 (2.96, 7.52)
Sleep duration*All other races	0.60 (0.29,1.25)	1.13 (0.72, 1.80)	1.70 (0.99, 2.91)	0.83 (0.16, 4.18)
Sleep duration*Black or African American	0.99 (0.32, 3.07)	0.89 (0.57, 1.38)	1.23 (0.69, 2.20)	0.75 (0.14, 3.97)
Sleep duration *Hispanic/Latino	0.85 (0.50, 1.43)	0.97 (0.72, 1.32)	0.92 (0.63, 1.34)	0.52 (0.15, 1.78)
Joint Test	Suicide attempt F test (p-value)	Considered suicide F test (p-value)	Made a suicide plan F test (p-value)	Injurious suicide attempt F test (p-value)
Sleep*race	0.71 (0.55)	0.21 (0.89)	1.60 (0.19)	0.38 (0.77)

**Fig. 2:**
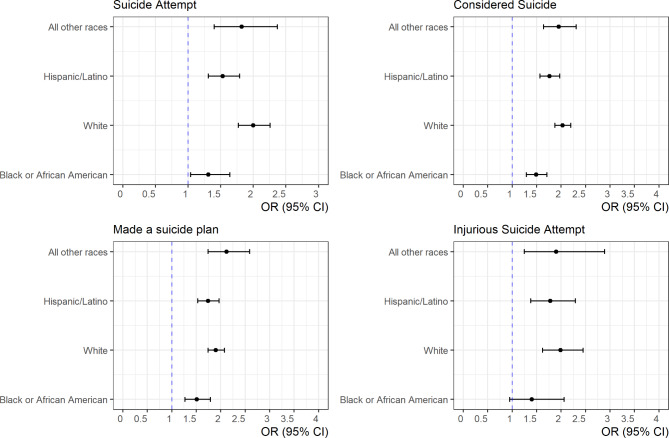
Odds ratios with 95% confidence intervals of sleep duration and suicide among each race/ethnicity

To better understand the direction and nature of this interaction, Fig. 2 presents odds ratios to measure the relationship between sleep duration and suicidal ideation/behavior stratified by race/ethnicity. Among all races, short sleep was significantly associated with suicide ideation/behavior. Black students with short sleep had higher odds of making a suicide plan (OR = 1.51, 95% C.I.: 1.27, 1.79) compared with Black students with long sleep. The relationship, however, was larger for other racial/ethnic groups: Hispanic (OR = 1.74, 95% C.I.: 1.53, 1.97), White (OR = 1.90, 95% C.I.: 1.74, 2.07) and “All other races” (OR = 2.12, 95% C.I.: 1.74, 2.59).

## Discussion

Two main findings emerged from this study (1) sleep duration is significantly associated with suicidal ideation/behavior among U.S. adolescents; (2) race/ethnicity modifies the association between sleep duration and suicidal ideation/behavior, with the magnitude of the association between sleep and suicidal behavior being generally lower for Black and Hispanic adolescents. These findings support previous findings [17] and highlight the importance of suicide related interventions to better adolescents’ health and the necessity to address poor sleep health among U.S. youth. Further, efforts to reduce suicidal behavior among Black and Hispanic adolescents should focus on additional mechanisms through which suicidal behavior arises.

Although sleep duration significantly predicts suicide ideation/behavior among all adolescents, the magnitude of the association is highest among adolescents in the “all other races” category. Nonetheless, the need to intervene against suicide among adolescents is clear. Beginning in 2007, suicide rates among adolescents in all racial groups have increased by 57.4% [1]. This high increase is of great concern. The smaller association between sleep and suicidality for Black and Hispanic students may be due to the underreporting of mental health outcomes among minoritized populations [22]. Hence, the magnitude of the association might be underestimated and Black/Hispanic students with short sleep with suicidal behaviors/thoughts may be misclassified as those without suicidal behaviors/thoughts. This indicates the need to focus on two aspects: (1) identifying additional predictors of suicide ideation/behavior among Black/African American and Hispanic/Latino adolescents and (2) identifying better screening tools for suicidal ideation/behaviors among Black/African American and Hispanic/Latino adolescents. The current literature indicates that minoritized groups who die by suicide are less likely to report previous suicidal ideation/attempts [23]. Without accurate assessments of suicide risk behavior among racial minorities, it is difficult to predict and prevent suicide and related health behaviors.

A sociological understanding of the construction of ‘race’ as a social category is paramount to improving population health and reduce disparities. Race is not a biological construct [24]. Rather, it is a social construct that captures the impacts and legacy of racism [25], including how science reifies race and ethnicity through broad categorization of racial and ethnic group membership rather than direct measures of racism experiences and history. Hence, through assessing racialized disparities in suicide, this study contributes to identifying the impact of racism on adolescent mental health outcomes, and race-specific interventions. It is important to understand trends by race to provide targeted and efficient interventions focused on the social determinants. The social environment influences health outcomes, and racialized disparities are a consequence of the legacy of historical racism that continues to differentially expose minoritized adolescents to adverse conditions that affect sleep and mental health.

Few suicide and mental health screening tools are specifically calibrated for Black and Hispanic adolescents. To our knowledge, current screening tools for the suicidality of Black and Hispanic adolescents include depression screeners such as the Patient Health Questionnaire (PHQ)-9 and PHQ-2 [26]. Sleep is a major pillar of health in addition to diet and exercise, especially but not only among adolescents. The public health implications of poor sleep health on adolescents are numerous and play a critical role not only in the short term but throughout the life course as well [27]. Our study shows that sleep health may contribute to the increases in suicidality among minoritized youth. Predictors of sleep health specifically among Black and Hispanic adolescents also urgently require additional research [28]. Documented predictors of sleep health among Black and Hispanic adolescents include daily discrimination and neighborhood safety [29]. Even though the predictors are systemic and structural, many of the interventions on sleep are at the individual level. Although those types of interventions have had successful results [30], they tend to not cater to the less privileged and/or not address structural issues that allow sleep disparities to persist. Some sleep interventions include sleep schedules, relaxation techniques, physical exercise, e-health interventions such as meditation with mobile applications, and psychotherapy. Individuals who do not have access to resources such as health insurance and time availability would not benefit from the individual-level interventions listed above. Less is known about sleep interventions among minoritized adolescents. Many adolescents do not get adequate sleep on an average school night due to the amount of schoolwork that needs to be completed in addition to participating in extracurricular activities. In certain communities, such as low-income communities, adolescents have additional tasks such as caring for younger siblings and ensuring that food is provided because their parents must work. More research is needed to determine successful, culturally competent sleep interventions among minoritized adolescents.

The association between sleep duration and suicide risk is clear [31]. Theoretical frameworks such as the interpersonal-psychological theory (IPTS) contribute to the understanding of suicide pathways. Specially, IPTS suggests that to want to die by suicide, an individual must perceive him or herself as a burden to others and feel a lack of belongingness and is at greatest risk of dying by suicide when combined with the ability to accomplish lethal self-harm [32]. Constructs of IPTS have been associated with sleep disorders [32] indicating that sleep duration may be a mediator in the causal pathway between IPTS constructs such has lack of belongingness and suicide. Hence, evidence-based resiliency programs with a focus on protective factors including sense of belonging are needed [33]. Moreover, interventions to increase mental health resource access among vulnerable groups are essential. Experiences of mental health and mental health care among minoritized populations shape individual perceptions of mental illness. Historically, racial minorities have been less likely to receive mental healthcare due to historical trauma from unfair practices in mental health, structural inequities, mistrust of health professionals, stigma, social determinants of health, and lack of awareness [34, 35]. The lack of mental health access among minority populations needs to be addressed and interventions need to be tailored depending on mental health background. This study’s findings also have clinical significance. Existing clinical suicide risk reduction strategies include safety planning and motivational interviewing, screening, and cognitive behavior therapy [36]. Thus, in addition to increasing mental health access, the effectiveness of clinical interventions among racial minority groups needs to be ensured. Little is known regarding the mechanisms that factor into the reduced likelihood for Black populations to report suicide ideation/attempt. Public health interventions on establishing trust between study participants and investigators are critical to reduce response bias and increase the likelihood of accurately measuring suicide risk among Black/Hispanic populations, thereby assessing a more accurate relationship between sleep health and suicide risk.

This study had limitations due to the structure of the YRBS. Not all national YRBS surveys could be used; questionnaires before 2007 did not include questions on sleep duration. Future studies should seek to assess longer-time trends on the impact of sleep duration on suicide risk. Due to race and ethnicity questions changing over time, the YRBS provided a 4 and 7-level indicator variable to bridge changes. This study uses the 4-level variable from the race and ethnicity questions which combines American Indian/Alaska Native (n = 650), Asian (n = 3193), Native Hawaiian/Other Pacific Islander (n = 610), and Multiple Races-non-Hispanic (n = 3951) into one overarching category. Although this limits the ability to assess trends among each of those groups and contributes to the statistical erasure of important subgroups, the overarching category was preferred to strengthen the reliability and validity of results considering the small sample sizes in each category. Wide confidence intervals indicate that statistical power may have been compromised. Future studies incorporating datasets with larger racial/ethnic data are needed. Responder bias may contribute to overreporting or underreporting of results since survey data is being used. Furthermore, future research should examine the impact of other measures of sleep health such as sleep disorders, sleep quality, and tiredness on suicide risk. Family socioeconomic status, an important variable associated with sleep and suicide, was not included as a covariate as it was not assessed in the YRBS. Lastly, YRBS administers surveys to students in private and public high schools. Therefore, adolescents who are not enrolled in high schools are not included in our sample. To our knowledge, this study is one of the first to assess the impact of sleep deprivation on suicide ideation/behavior among a large sample of U.S. adolescents. It also contributes to a growing body of work assessing specific factors that impact suicidality among minoritized adolescents. Future research should continue to explore race-specific predictors of suicide among U.S. adolescents.

## Conclusions

This study indicates the need to implement additional screening tools to assess suicidal ideation/outcomes among minoritized youth. Emphasis on suicide interventions is of the essence, especially with increasing rates. Sleep duration significantly predicts suicide risk among all adolescents. Additional research is needed to assess factors that predict suicide ideation/behavior among minoritized adolescents, specifically Black and Hispanic adolescents.

### Electronic supplementary material

Below is the link to the electronic supplementary material.

Supplemental Tables and Figures

## Data Availability

The datasets generated and/or analyzed during the current study are available in the Combined YRBS High School Datasets and Documentation repository, https://www.cdc.gov/healthyyouth/data/yrbs/data.htm.
