# Supplementary material for: Sleep deprivation and suicide risk among minoritized US adolescents
Source: BMC Psychiatry. 2023 Aug 31;23:638. doi: 10.1186/s12888-023-05074-3 (PMC10472686; doi:10.1186/s12888-023-05074-3)

**Supplemental Tables and Figures**

Table 1. N (%) of study participants in each independent variable by two categories of suicide attempt

|  | Total | Attempted suicide, N (%) | Did not attempt suicide, N (%) | Rao-Scott Chi-Square test statistic (p-value) |
| --- | --- | --- | --- | --- |
| N (%) | 86362 | 7,356 (7.65) | 79,006 (92.35) |  |
|  |  |  |  |  |
| Sleep duration |  |  |  | 179.73 (<.0001) |
| Less than 8 hours | 64,995 (71.50) | 5,287 (81.19) | 51,162 (71.07) |  |
| 8 hours or more | 25,924 (28.50) | 1,242 (18.81) | 20,761 (28.93) |  |
| Missing | 12,606 |  |  |  |
|  |  |  |  |  |
| Race |  |  |  | 193.83 (<.0001) |
| White | 44,062 (55.88) | 2,555 (47.56) | 35,682 (59.22) |  |
| Black or African American | 18,026 (13.89) | 1,263 (14.11) | 12,213 (12.03) |  |
| Hispanic/Latino | 28,463 (21.042) | 2,377 (25.95) | 21,551 (19.84) |  |
| All other races | 10,659 (9.19) | 942 (12.37) | 8,069 (8.92) |  |
| Missing | 2,315 |  |  |  |
|  |  |  |  |  |
| Sexual Identity |  |  |  | 894.03 (<.0001) |
| Heterosexual | 35,819 (86.28) | 1,860 (63.61) | 25,625 (88.50) |  |
| Gay or Lesbian | 1,061 (2.29) | 163 (5.29) | 614 (1.94) |  |
| Bisexual | 3,210 (7.51) | 679 (24.52) | 1,862 (6.13) |  |
| Not Sure | 1,696 (3.92) | 213 (6.58) | 1,005 (3.43) |  |
| Missing | 61,739 |  |  |  |
|  |  |  |  |  |
| Age |  |  |  | 25.22 (<.0001) |
| 12 -15 years old | 36,028 (36.51) | 2,789 (40.06) | 26,747 (36.28) |  |
| 16 years old or older | 67,008 (63.49) | 4,522 (59.94) | 51,938 (63.72) |  |
| Missing | 489 |  |  |  |
|  |  |  |  |  |
| Sex |  |  |  | 240.37 (<.0001) |
| Female | 51,813 (49.16) | 4,797 (64.74) | 39,037 (48.55) |  |
| Male | 51,165 (50.84) | 2,475 (35.26) | 39,669 (51.45) |  |
| Missing | 547 |  |  |  |
|  |  |  |  |  |
| Alcohol Use |  |  |  | 622.65 (<.0001) |
| No drink in past 30 days | 59,602 (63.88) | 2,616 (41.35) | 47,218 (65.21) |  |
| Drink at least one day in the past 30 days | 33,871 (36.12) | 3,618 (58.65) | 25,485 (34.79) |  |
| Missing | 10,052 |  |  |  |
|  |  |  |  |  |
| Marijuana Use |  |  |  | 1122.78 (<.0001) |
| No marijuana uses over past 30 days | 78,773 (78.55) | 4,097 (57.05) | 62,214 (80.55) |  |
| Marijuana use at least once in the past 30 days | 22,228 (21.45) | 2,891 (42.94) | 15,685 (19.45) |  |
| Missing | 2,524 |  |  |  |
|  |  |  |  |  |
| Cocaine Use |  |  |  | 1644.39 (<.0001) |
| No uses of any form of cocaine in life | 94,461 (94.26) | 56,70 (80.06) | 73,565 (95.69) |  |
| Used a form of cocaine at least once in life | 6,242 (5.73) | 1402 (19.94) | 3629 (4.31) |  |
| Missing | 2,822 |  |  |  |
|  |  |  |  |  |
| Trauma |  |  |  | 1907.63 (<.0001) |
| Yes | 791 (1.70) | 336 (14.93) | 308 (0.88) |  |
| No | 44,003 (98.30) | 1,955 (85.07) | 34,314 (99.12) |  |
| Missing | 58,731 |  |  |  |
|  |  |  |  |  |
| Bullying |  |  |  | 1579.22 (<.0001) |
| Yes | 6,462 (11.56) | 1,423 (42.06) | 3,963 (9.59) |  |
| No | 53,505 (88.44) | 2,328 (57.94) | 41,034 (90.41) |  |
| Missing | 43,558 |  |  |  |

Supplemental Table 2. N (%) of study participants in each independent variable by two categories of suicide consideration

|  | Total | Considered suicide, N (%) | Did not consider suicide, N (%) | Rao-Scott Chi-Square test statistic (p-value) |
| --- | --- | --- | --- | --- |
| N (%) | 102,287 | 17,136 (16.34) | 85,151 (83.66) |  |
|  |  |  |  |  |
| Sleep duration |  |  |  | 495.09 (<.0001) |
| Less than 8 hours | 64,995 (71.50) | 12256 (81.15) | 52,161 (69.54) |  |
| 8 hours or more | 25,924 (28.50) | 2,924 (18.85) | 22,837 (30.46) |  |
| Missing | 12,606 |  |  |  |
|  |  |  |  |  |
| Race |  |  |  | 84.28 (<.0001) |
| White | 44,062 (55.88) | 7,280 (54.56) | 36,345 (56.22) |  |
| Black or African American | 18,026 (13.89) | 2,548 (12.02) | 15,252 (14.22) |  |
| Hispanic/Latino | 28,463 (21.042) | 4,785 (22.03) | 23,351 (20.82) |  |
| All other races | 10,659 (9.19) | 2,112 (11.38) | 8,410 (8.74) |  |
| Missing | 2,315 |  |  |  |
|  |  |  |  |  |
| Sexual Identity |  |  |  | 1429.64 (<.0001) |
| Heterosexual | 35,819 (86.28) | 5,189 (68.35) | 30,243 (90.35) |  |
| Gay or Lesbian | 1,061 (2.29) | 387 (4.92) | 633 (1.69) |  |
| Bisexual | 3,210 (7.51) | 1,506 (19.98) | 1,643 (4.72) |  |
| Not Sure | 1,696 (3.92) | 538 (6.74) | 1,111 (3.24) |  |
| Missing | 61,739 |  |  |  |
|  |  |  |  |  |
| Age |  |  |  | 1.28 (0.26) |
| 12 -15 years old | 36,028 (36.51) | 6,199 (36.90) | 29,287 (36.29) |  |
| 16 years old or older | 67,008 (63.49) | 10,850 (63.10) | 55,488 (63.71) |  |
| Missing | 489 |  |  |  |
|  |  |  |  |  |
| Sex |  |  |  | 599.33 (<.0001) |
| Female | 51,813 (49.16) | 11,054 (63.39) | 40,233 (46.43) |  |
| Male | 51,165 (50.84) | 5,946 (36.61) | 44,565 (53.57) |  |
| Missing | 547 |  |  |  |
|  |  |  |  |  |
| Alcohol Use |  |  |  | 670.47 (<.0001) |
| No drink in past 30 days | 59,602 (63.88) | 7,538 (48.72) | 51,591 (66.76) |  |
| Drink at least one day in the past 30 days | 33,871 (36.12) | 7,668 (51.28) | 25,896 (33.24) |  |
| Missing | 10,052 |  |  |  |
|  |  |  |  |  |
| Marijuana Use |  |  |  | 1028.74 (<.0001) |
| No marijuana uses over past 30 days | 78,773 (78.55) | 11,029 (65.76) | 67,113 (81.10) |  |
| Marijuana use at least once in the past 30 days | 22,228 (21.45) | 5,596 (34.24) | 16,355 (18.90) |  |
| Missing | 2,524 |  |  |  |
|  |  |  |  |  |
| Cocaine Use |  |  |  | 1162.51 (<.0001) |
| No uses of any form of cocaine in life | 94,461 (94.26) | 14,534 (87.39) | 79,161 (95.70) |  |
| Used a form of cocaine at least once in life | 6,242 (5.73) | 2,139 (12.61) | 3,910 (4.30) |  |
| Missing | 2,822 |  |  |  |
|  |  |  |  |  |
| Trauma |  |  |  | 742.56 (<.0001) |
| Yes | 791 (1.70) | 490 (7.40) | 291 (0.73) |  |
| No | 44,003 (98.30) | 5,942 (92.59) | 37,780 (99.27) |  |
| Missing | 58,731 |  |  |  |
|  |  |  |  |  |
| Bullying |  |  |  | 3366.79 (<.0001) |
| Yes | 6,462 (11.56) | 3,005 (34.01) | 3,409 (7.45) |  |
| No | 53,505 (88.44) | 6,264 (65.99) | 47,028 (92.55) |  |
| Missing | 43,558 |  |  |  |

Supplemental Table 3. N (%) of study participants in each independent variable by two categories of suicide plan

|  | Total | Made a suicide plan, N (%) | Did not make a suicide plan, N (%) | Rao-Scott Chi-Square test statistic (p-value) |
| --- | --- | --- | --- | --- |
| N (%) | 101,882 | 13,922 (13.15) | 87,960 (86.85) |  |
|  |  |  |  |  |
| Sleep duration |  |  |  | 392.93 (<.0001) |
| Less than 8 hours | 64,995 (71.50) | 9,944 (81.05) | 54,229 (69.97) |  |
| 8 hours or more | 25,924 (28.50) | 2,349 (18.95) | 23,322 (30.03) |  |
| Missing | 12,606 |  |  |  |
|  |  |  |  |  |
| Race |  |  |  | 85.97 (<.0001) |
| White | 44,062 (55.88) | 5,743 (53.28) | 37,836 (56.40) |  |
| Black or African American | 18,026 (13.89) | 2,045 (12.28) | 15,666 (14.06) |  |
| Hispanic/Latino | 28,463 (21.042) | 4,008 (22.83) | 24,033 (20.78) |  |
| All other races | 10,659 (9.19) | 1,763 (11.60) | 8,617 (8.76) |  |
| Missing | 2,315 |  |  |  |
|  |  |  |  |  |
| Sexual Identity |  |  |  | 1400.09 (<.0001) |
| Heterosexual | 35,819 (86.28) | 4,142 (67.53) | 31,073 (89.69) |  |
| Gay or Lesbian | 1,061 (2.29) | 334 (4.94) | 681 (1.80) |  |
| Bisexual | 3,210 (7.51) | 1,293 (20.79) | 1,830 (5.15) |  |
| Not Sure | 1,696 (3.92) | 423 (6.73) | 1,210 (3.36) |  |
| Missing | 61,739 |  |  |  |
|  |  |  |  |  |
| Age |  |  |  | 0.42 (0.51) |
| 12 -15 years old | 36,028 (36.51) | 5,068 (36.72) | 30,297 (36.33) |  |
| 16 years old or older | 67,008 (63.49) | 8,774 (63.28) | 57,283 (63.67) |  |
| Missing | 489 |  |  |  |
|  |  |  |  |  |
| Sex |  |  |  | 384.09 (<.0001) |
| Female | 51,813 (49.16) | 8,659 (61.44) | 42435 (47.34) |  |
| Male | 51,165 (50.84) | 5,143 (38.56) | 45,162 (52.66) |  |
| Missing | 547 |  |  |  |
|  |  |  |  |  |
| Alcohol Use |  |  |  | 651.13 (<.0001) |
| No drink in past 30 days | 59,602 (63.88) | 6,079 (48.73) | 52,821 (66.09) |  |
| Drink at least one day in the past 30 days | 33,871 (36.12) | 6,191 (51.27) | 27,258 (33.91) |  |
| Missing | 10,052 |  |  |  |
|  |  |  |  |  |
| Marijuana Use |  |  |  | 924.56 (<.0001) |
| No marijuana uses over past 30 days | 78,773 (78.55) | 8,810 (64.84) | 69,032 (80.66) |  |
| Marijuana use at least once in the past 30 days | 22,228 (21.45) | 4,651 (35.16) | 17,226 (19.33) |  |
| Missing | 2,524 |  |  |  |
|  |  |  |  |  |
| Cocaine Use |  |  |  | 970.62 (<.0001) |
| No uses of any form of cocaine in life | 94,461 (94.26) | 11,654 (86.43) | 81,690 (95.55) |  |
| Used a form of cocaine at least once in life | 6,242 (5.73) | 1877 (13.57) | 4,133 (4.45) |  |
| Missing | 2,822 |  |  |  |
|  |  |  |  |  |
| Trauma |  |  |  | 872.05 (<.0001) |
| Yes | 791 (1.70) | 450 (8.70) | 324 (0.76) |  |
| No | 44,003 (98.30) | 4,740 (91.30) | 38,776 (99.24) |  |
| Missing | 58,731 |  |  |  |
|  |  |  |  |  |
| Bullying |  |  |  | 2102.28 (<.0001) |
| Yes | 6,462 (11.56) | 2,479 (34.35) | 3,906 (8.30) |  |
| No | 53,505 (88.44) | 5,095 (65.65) | 47,953 (91.70) |  |
| Missing | 43,558 |  |  |  |

Supplemental Table 4. N (%) of study participants in each independent variable by two categories of injurious suicide attempt

|  | Total | Injurious attempt suicide, N (%) | No injurious suicide attempt, N (%) | Rao-Scott Chi-Square test statistic (p-value) |
| --- | --- | --- | --- | --- |
| N (%) | 83,249 | 2,204 (2.38) | 81,045 (97.62) |  |
|  |  |  |  |  |
| Sleep duration |  |  |  | 79.65 (<.0001) |
| Less than 8 hours | 64,995 (71.50) | 1,576 (82.49) | 52,672 (71.52) |  |
| 8 hours or more | 25,924 (28.50) | 359 (17.51) | 20,967 (28.48) |  |
| Missing | 12,606 |  |  |  |
|  |  |  |  |  |
| Race |  |  |  | 88.09 (<.0001) |
| White | 44,062 (55.88) | 746 (45.44) | 36,141 (58.75) |  |
| Black or African American | 18,026 (13.89) | 374 (14.65) | 12,360 (12.01) |  |
| Hispanic/Latino | 28,463 (21.042) | 726 (27.15) | 22,654 (20.20) |  |
| All other races | 10,659 (9.19) | 287 (12.76) | 8,379 (9.04) |  |
| Missing | 2,315 |  |  |  |
|  |  |  |  |  |
| Sexual Identity |  |  |  | 253.04 (<.0001) |
| Heterosexual | 35,819 (86.28) | 527 (62.81) | 25,382 (87.13) |  |
| Gay or Lesbian | 1,061 (2.29) | 47 (5.57) | 645 (2.07) |  |
| Bisexual | 3,210 (7.51) | 201 (24.15) | 2160 (7.25) |  |
| Not Sure | 1,696 (3.92) | 61 (7.46) | 1057 (3.55) |  |
| Missing | 61,739 |  |  |  |
|  |  |  |  |  |
| Age |  |  |  | 5.72 (0.02) |
| 12 -15 years old | 36,028 (36.51) | 812 (39.52) | 27,496 (36.46) |  |
| 16 years old or older | 67,008 (63.49) | 1,378 (60.48) | 53, 212 (63.54) |  |
| Missing | 489 |  |  |  |
|  |  |  |  |  |
| Sex |  |  |  | 76.94 (<.0001) |
| Female | 51,813 (49.16) | 1,405 (63.60) | 40,844 (49.45) |  |
| Male | 51,165 (50.84) | 772 (36.40) | 39892 (50.55) |  |
| Missing | 547 |  |  |  |
|  |  |  |  |  |
| Alcohol Use |  |  |  | 476.31 (<.0001) |
| No drink in past 30 days | 59,602 (63.88) | 567 (29.92) | 47,374 (64.16) |  |
| Drink at least one day in the past 30 days | 33,871 (36.12) | 1,260 (70.08) | 26,983 (35.84) |  |
| Missing | 10,052 |  |  |  |
|  |  |  |  |  |
| Marijuana Use |  |  |  | 643.82 (<.0001) |
| No marijuana uses over past 30 days | 78,773 (78.55) | 964 (45.68) | 63,148 (79.63) |  |
| Marijuana use at least once in the past 30 days | 22,228 (21.45) | 1,084 (54.32) | 16,716 (20.36) |  |
| Missing | 2,524 |  |  |  |
|  |  |  |  |  |
| Cocaine Use |  |  |  | 1679.04 (<.0001) |
| No uses of any form of cocaine in life | 94,461 (94.26) | 1,437 (68,13) | 75,611 (95.16) |  |
| Used a form of cocaine at least once in life | 6,242 (5.73) | 657 (31.87) | 4,171 (4.84) |  |
| Missing | 2,822 |  |  |  |
|  |  |  |  |  |
| Trauma |  |  |  | 1537.74 (<.0001) |
| Yes | 791 (1.70) | 174 (27.49) | 457 (1.27) |  |
| No | 44,003 (98.30) | 444 (72.50) | 34,801 (98.73) |  |
| Missing | 58,731 |  |  |  |
|  |  |  |  |  |
| Bullying |  |  |  | 852.58 (<.0001) |
| Yes | 6,462 (11.56) | 532 (50.21) | 4569 (11.07) |  |
| No | 53,505 (88.44) | 576 (49.79) | 40,718 (88.93) |  |
| Missing | 43,558 |  |  |  |

Supplemental Table 5. Logistic regression assessing the relationship between sleep duration and suicide

|  | Suicide attempt vs. no OR (95% CI) | Considered suicide vs. no OR (95% CI) | Made a suicide plan vs. no  OR (95% CI) | Injurious suicide attempt vs. no  OR (95% CI) |
| --- | --- | --- | --- | --- |
| Sleep Duration |  |  |  |  |
| 8 hours of sleep or more | Ref | Ref | Ref | Ref |
| Less than 8 hours of sleep | 1.76 (1.61, 1.91) | 1.89 (1.78, 2.00) | 1.84 (1.73, 1.95) | 1.88 (1.63, 2.16) |

*Results show odds ratios and 95% confidence intervals from unadjusted logistic regression output assessing the relationship between sleep duration and suicide.

Supplemental Table 6. Multivariable logistic regression assessing the relationship between sleep duration and suicide and interactions with sleep duration and race/ethnicity (parameter estimates and p-values)

|  | Suicide attempt vs. no | Considered suicide vs. no | Made a suicide plan vs. no | Injurious suicide attempt vs. no |
| --- | --- | --- | --- | --- |
| Sleep duration (ref=  8 hours of sleep or more) |  |  |  |  |
| Less than 8 hours of sleep | 0.69 (<.0001) | 0.71 (<.0001) | 0.64 (<.0001) | 0.70 (<.0001) |
|  |  |  |  |  |
| Race (ref=White) |  |  |  |  |
| All other races | 0.60 (<.0001) | 0.32 (0.0004) | 0.24 (.0.02) | 0.60 (0.01) |
| Black or African American | 0.67 (<.0001) | 0.11 (0.15) | 0.10 (0.30) | 0.65 (0.001) |
| Hispanic/Latino | 0.69 (<.0001) | 0.21 (0.002) | 0.23 (0.001) | 0.65 (<.0001) |
|  |  |  |  |  |
| Sleep duration*All other races | -0.09 (0.55) | -0.04 (0.66) | 0.11 (0.34) | -0.05 (0.84) |
| Sleep duration*Black or African American | -4.42 (0.001) | -0.31 (0.0003) | -0.23 (0.02) | -0.35 (0.100) |
| Sleep duration *Hispanic/Latino | -0.27 (0.01) | -0.15 (0.04) | -0.09 (0.24) | -0.11 (0.47) |
|  |  |  |  |  |
| Joint Test | Suicide attempt F test (p-value) | Considered suicide F test (p-value) | Made a suicide plan F test (p-value) | Injurious suicide attempt F test (p-value) |
| Sleep*race | 4.88 (0.002) | 5.40 (0.001) | 2.86 (0.04) | 1.05 (0.37) |

Supplemental Figure 1. Prevalence of suicide by race among US youth, 2007-2019


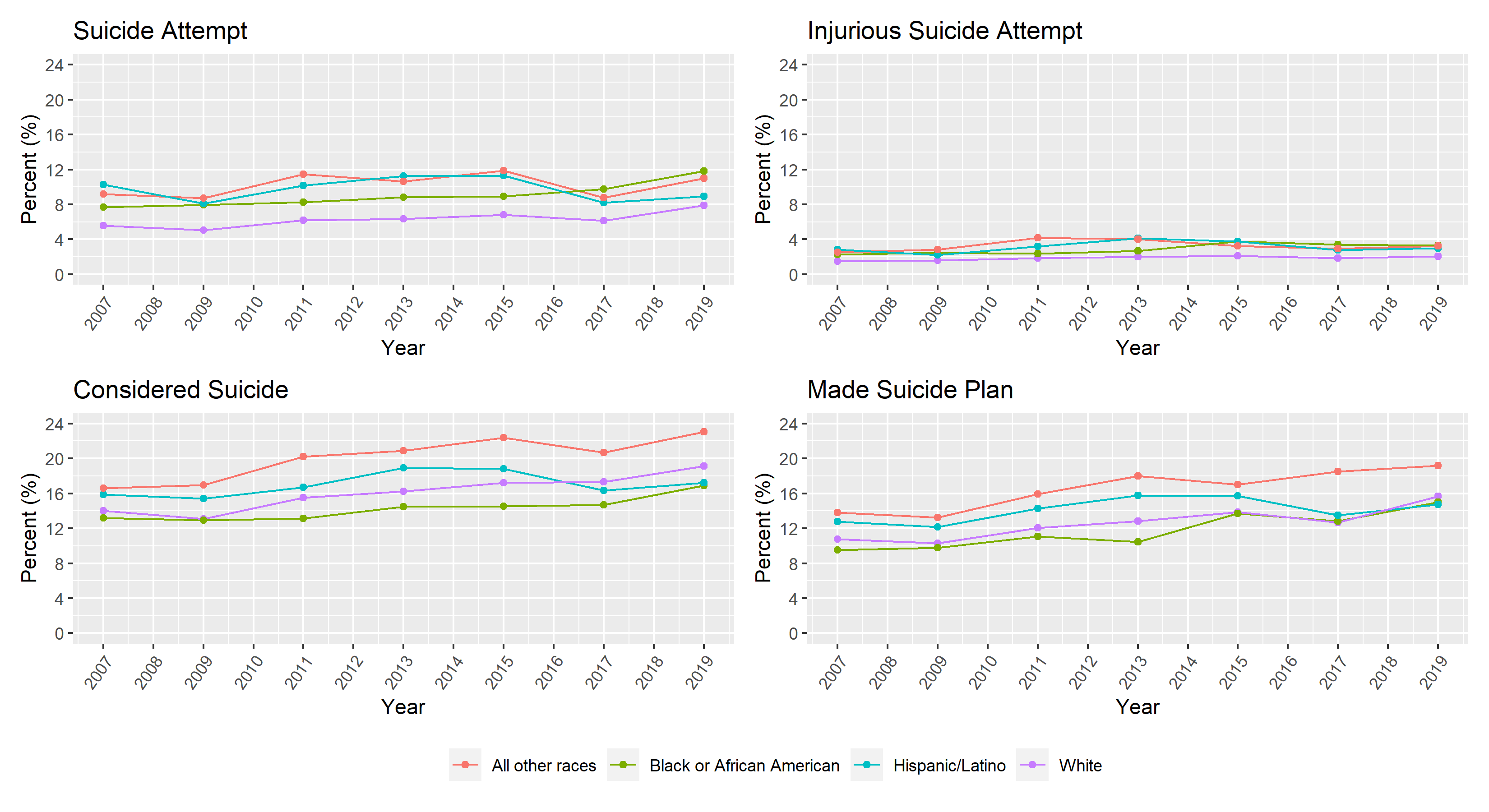

Supplement: Supplementary file 1 — Supplemental Tables and Figures [file 12888_2023_5074_MOESM1_ESM.docx]
